# Resilience Moderates Negative Outcome from Stress during the COVID-19 Pandemic: A Moderated-Mediation Approach

**DOI:** 10.3390/ijerph17186461

**Published:** 2020-09-04

**Authors:** Audun Havnen, Frederick Anyan, Odin Hjemdal, Stian Solem, Maja Gurigard Riksfjord, Kristen Hagen

**Affiliations:** 1 Department of Psychology, Norwegian University of Science and Technology, 7491 Trondheim, Norway; frederick.anyan@ntnu.no (F.A.); odin.hjemdal@ntnu.no (O.H.); stian.solem@ntnu.no (S.S.); 2 Molde Hospital, Møre og Romsdal Hospital Trust, 6412 Molde, Norway; maja.gurigard.riksfjord@helse-mr.no (M.G.R.); kristen.hagen@helse-mr.no (K.H.); 3 Department of Mental Health, Norwegian University of Science and Technology, 7491 Trondheim, Norway

**Keywords:** stress, anxiety, depression, resilience, moderated mediation

## Abstract

Resilience refers to an individual’s healthy coping abilities when encountering adverse life events. The COVID-19 pandemic represents a situation with a high amount of stress exposure, which in turn may be associated with negative emotional outcome like depressive symptoms. The current study investigated if resilience moderated the effect of stress on symptoms of depression and if anxiety symptoms mediated this association. An adult sample of community controls completed the Perceived stress scale 14 (PSS-14), the Resilience scale for adults (RSA), the Patient health questionnaire 9 (PHQ-9) and the Generalized anxiety disorder 7 (GAD-7). Independent samples t-test, correlation analyses and moderated mediation analyses were conducted. The results showed that resilience moderated the relations between stress and anxiety symptoms (β = −0.131, *p* < 0.001) as well as between stress and depressive symptoms (β = −0.068, *p* < 0.05). In support of a moderated mediation model, resilience moderated the indirect effect of stress on depressive symptom, as confirmed by the index of moderated mediation (IMM = −0.036, *p* < 0.001; [95% BCa: −0.055, −0.020]). The high resilience subgroup was less affected than the low resilience subgroup by the effect of stress exposure symptoms of depression, mediated by anxiety. The study shows that stress exposure is associated with symptoms of depression, and anxiety mediates this association. Level of resilience differentiates the direct and indirect effect of stress on depression. Knowledge about the effect of stress in response to a pandemic is important for developing treatment and prevention strategies for stress, depression and health-related anxiety.

## 1. Introduction

The outbreak of coronavirus disease 2019 (COVID-19) in late 2019 and early 2020 caused major global concerns. Since the virus was first identified, it rapidly spread throughout the world. In many countries, people directly exposed to the coronavirus and those who had been in contact with infected people were instructed to isolate themselves. Many countries also closed their national borders to restrict international travel and practiced a broad shut-down for their populations. A recent review paper showed that quarantine is associated with negative psychological consequences, like symptoms of post-traumatic stress disorder and anger [1]. Mass-media covered the COVID-19 extensively and studies suggest that media coverage may also contribute to increased fear among people [2]. WHO termed the Corona virus ‘public enemy number 1′ [3]. With such descriptions from health authorities combined with the massive restrictions many countries implemented, it might be expected that the COVID-19 represents a significant stressor in persons’ daily lives.

It has previously been documented that publicizing disease outbreaks can lead to increased health anxiety even among people that are medically healthy [4]. Increased levels of anxiety and depression have been reported in association with pandemic situations like the outbreak of SARS [5,6], the Asian flu [7], and the swine flu [8]. Knowledge about psychological factors that moderate negative outcome from stress and anxiety in response to a pandemic is essential, due to the relevance for treatment and prevention strategies for stress, depression and health-related anxiety [9].

According to the transactional theory developed by Lazarus and Folkman [10], the degree to which a person believes he or she has the resources needed to cope with a situation determines whether the situation will be experienced as a stressor, and a perceived lack of resources will significate a stressful event. In other words, the phenomenon of stress is understood as a subjective experience dependent upon perceived resources and demands. In stress research there has been a shift from investigating potential vulnerability factors over to the inherent ability many people seemingly have, that helps them adapt to difficult situations [11].

Factors that contribute to healthy coping and health promotion have been termed ‘resilience’, which is a description of healthy adaptation despite adversity. There are a great number of factors and protective processes linked both to personal attributes and skills, as well as supportive social environment that contribute to resilient behavior. Resilient individuals are characterized by a variety of inter- and intrapersonal characteristics which, also involve a higher degree of flexibility, and to apply more appropriate coping strategies than less resilient persons [12,13]. The protective role of resilient factors is supported by a range of research, spanning over four decades [14,15,16]. A resilience framework specifies protective factors and processes and addresses how these contribute to both main and buffering effects in health. The Reserve Capacity Model [17] is a relevant model that fits particularly well with a resilience perspective with regards to buffering effects as it highlights how the protective factors may moderate and mediate outcome. However, the field of resilience research is not represented by an overarching and unifying theoretical framework and the causal trajectories are debated [18].

Previous research on resilience have suffered from methodological issues such as unclear definitions of resilience, and use of instruments with unknown psychometric properties [19]. The Resilience Scale for Adults (RSA) [20,21] was developed to overcome such limitations. The RSA is a 33-item questionnaire [22] that has been translated into various languages with good psychometric properties and predictive abilities [23]. For the total RSA score there are no sex differences, but there are sex differences in relation to the factor scores which are consistent with sex differences reported in other studies [24,25,26,27]. Studies have found that women generally report higher scores on social competency and occasionally on access to social resources while men report higher scores on perception of self which is related to believing in their own ability [20,28]. These sex differences may have clinical implications, as they imply that men and women may have difference preferences when working on improving resilience skills.

In stress research, resilience has been associated with a number of positive outcomes. Specifically, a higher level of resilience protects against adverse effects of stress exposure. For example, resilience is a better predictor for hopelessness than stressful life events, mood, and personality traits (measured with the full NEO-PI-R) [29]. Resilience also protects against traumatic stress and symptoms of depression in soldiers exposed to military operations [30]. Resilience is found to be a protective factor also for adolescents’ mental health. Resilient adolescents were found to be less vulnerable for symptoms of depression, anxiety, stress and obsessive-compulsive disorder [31].

Resilience has been found to buffer against stress during previous virus outbreaks. A study found that among patients who survived the SARS epidemic, resilient individuals had lower levels of SARS-related worry [32]. Gender differences were identified, with males showing higher levels of resilience. In a review paper on how societies should prepare for major disasters, factors to improve resilience were highlighted [15]. The authors concluded that for the outbreak of flu pandemics, action must be taken to promote resilience, for example by using electronic communication to promote family and social support. The promotion of resilience has therefore been suggested as an essential means of improving how individuals cope during the COVID−19 pandemic [33]. 

Resilience has been studied as a moderator of the effect of stress exposure and negative outcome. A moderator can be understood as characteristics that influence the relationship between the stressor and the negative outcome. Examples of moderators are demographic variables like gender and age, or psychological coping resources like levels of resilience factors. Mediators, on the other hand, can be conceptualized as processes that explain the relationship between the stressor and the negative outcome, such as psychological processes. Studies have suggested that resilience factors may have differentiated protective effect depending of the level of protective resources [34], which may be studied by means of moderated-mediation analysis [35,36,37]. This refers to how a mediation effect of exposure to stress systematically varies, depending on the scores of another variable, e.g., level of social support. 

An example is the mediating effect of anxiety on the relationship between stress exposure and depression depends on the level of resilience. In a study of potential mediators of negative outcome following stress exposure, it was found that symptoms of generalized anxiety disorder mediated the effect of stress on depression [38]. The authors suggested that exposure to stressful life events may evoke anxiety related cognitions, which in turn lead to depressive symptoms. Further, it was demonstrated that level of resilience varied systematically with the negative outcome. Specifically, the study showed a significant direct effect of stress on depression and a significant indirect effect of stress on depression mediated by anxiety, and that individuals high on resilience were less affected by negative outcome from exposure to stressors than persons low levels of resilience. 

Another study by the same research group [34] found that state anxiety mediated the relationship between exposure to stressors and negative outcome measured by depressive symptoms. The study showed that there was a differentiated moderating effect of resilience, with high resilience associated with less negative outcome than low resilience. The authors concluded that resilience buffers both direct negative effects of stress exposure as well as indirect negative effects.

The COVID-19 has potentially severe negative consequences both at the individual and societal level. Studies report that the outbreak of COVID-19 was associated with increased levels of stress, anxiety and depression [39]. At the population level, world-leading mental health experts have warned about the possible negative effects on mental health caused by physical distancing measures and social isolation for vulnerable groups [40]. Economical issues are a common cause of worry for many people, and the financial consequences of COVID-19 with reduced activity in many sectors, will influence the labour market and increase levels of unemployment, which is likely to cause psychological distress [41]. Hence, to study if resilience factors protect individuals from suffering adverse emotional outcome during the COVID-19 pandemic has important implications for mental health.

**Figure 1:**
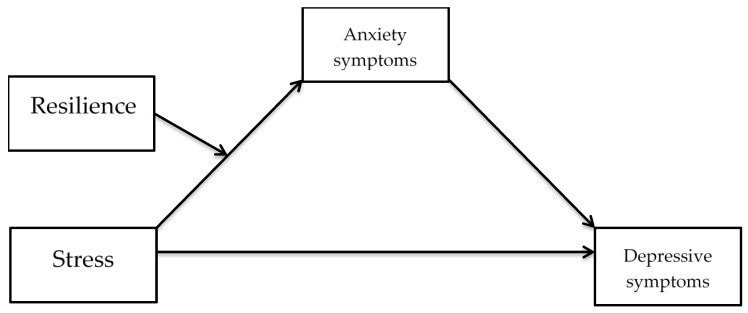
A conceptual diagram of hypothesized relations in a moderated mediation model.

With respect to these potentially negative consequences associated with the COVID-19, the current study aimed to investigate if resilience factors moderated symptoms of depression following exposure to stressors during the COVID-19 pandemic. We also aimed to study if generalized anxiety symptoms mediated the effect of stress on negative emotional outcome. Please refer to Figure 1 for a conceptual diagram of the hypothesized relations in a moderated mediation model. The present study may yield important information with a potential for targeting clinical implications aimed at enhancing resilience in the general public. 

### Hypotheses

**Hypothesis** **(H1).** *We expect symptoms of anxiety to mediate the relations between exposure to stress and depressive symptoms.*

**Hypothesis** **(H2).** *Resilience will moderate both the direct effect of exposure to stress on depressive symptoms, and the indirect effect of exposure to stress through anxiety symptoms on depressive symptoms.*

## 2. Materials and Methods 

### 2.1. Participants 

The study was formally reported to The Norwegian Centre for Research Data (reference number 2020/789067) who decided that the study did not need formal approval since the data collection was completely anonymous, in accordance with Norwegian legislation. The study did not need approval from the Regional ethics committee due to the non-identifying data collected. Participants were provided with contact information to the first author and the data protection officer at the university responsible for the study, if the survey raised any concern. Participants were invited for participation in the survey via an online link that was shared through social media. Norwegian voluntary mental health organizations helped spread the link to the survey through social media groups on Facebook or Instagram. Participants were required to be at least 18 years old and consent to participation. It was expected that a study with explicit reference to the COVID-19 pandemic may be vulnerable for response bias as individuals who are more concerned by the pandemic may be more likely to participate. To avoid this kind of response bias the survey did not include any reference to the COVID-19 pandemic, but was distributed in March 2020 at a point where the spread of the COVID-19 caused major restrictions in the Norwegian society. For example, all schools, university campuses and kindergartens were closed down, all employees who could work from home were instructed to do so, public transportation was encouraged not to be used, everyone (except core family members) was instructed to keep distance, all public areas were to be avoided. 

For registration of demographic variables, respondents were asked to provide information about gender (male, female, other), level of education and if they were working, student, on sick leave, disability benefit, retired or without work/at home. One question on lifetime history of mental illness was asked: “Have you ever been diagnosed with a mental disorder?” (yes or no). Education was coded on a 1–6 scale with 1 = Primary school, 2–3 lower or upper secondary school, 4 = Technical college, 5–6 = University/college below or above 4 years. 

### 2.2. Measures

Perceived Stress Scale 14 (PSS-14) [42] measures if the respondent has experienced stressful life events during the past month. The measure includes 14 items that are scored on a five-point Likert-scale from 0 (Never) to 4 (Very often). Total score ranges from 0–56 and is obtained by adding scores of all items. Higher scoring indicates more perceived stress. The PSS 14 is a valid measure with good psychometric properties [43,44]. Cronbach’s alpha in the present study was 0.89.

Resilience Scale for Adults (RSA) [20,21] is a 33-item self-report questionnaire that measures resilience; i.e., an individual’s protective resources when encountering life adversities. Items are scored on a 1–7 Likert scale. The RSA has a six-factor structure: (1) Perception of self, (2) Planned future, (3) Social competence, (4) Structured style, (5) Family cohesion and (6) Social resources. Total score range is 33 to 231; higher scoring indicates more protective resources. The RSA has good psychometric properties [28,45]. In the current study, Cronbach’s alpha was 0.93.

Generalized Anxiety Disorder 7 (GAD-7) [46] is a seven-item self-report questionnaire that was originally developed to measure symptoms of generalized anxiety disorder. Items are scored on a 0 (not at all) to 3 (almost every day) Likert scale. Higher total score equals more severe symptoms. GAD7 is also applicable to assess symptom severity of anxiety disorders in general [47]. A cut-off score at or above 10 is recommended to indicate the presence of an anxiety disorder. The GAD-7 has good psychometric properties [48]. Cronbach’s alpha was 0.87 in the current study.

Patient Health Questionnaire 9 (PHQ-9) [49] is a self-report questionnaire that measures symptoms of major depressive disorder. PHQ contains nine items that assess severity of different depressive symptoms and items are rated on a four-point Likert scale from 0 (not at all) to 3 (almost every day). Higher scoring indicates higher symptom severity and a total PHQ score at 10 or above is indicative of major depressive disorder. Cronbach’s alpha in this study was 0.89. 

### 2.3. Statistical Analyses 

Data preparation and initial statistical analyses were conducted in IBM SPSS version 25 (IBM Corporation, New York, NY, USA). Potential group differences for gender and mental illness were analyzed with t-tests. Levene’s tests for equality of variances were not significant for any of the analyses, therefore the assumption of equal variances was not violated. In order to reduce the risk of Type 1 error, the significance level was set to <0.01. Pearson correlation analysis was conducted to explore associations between continuous variables. Spearman’s rank correlations were conducted to investigate the relationship between categorical variables (gender, mental illness, work status and education) and continuous variables. The relationship between categorical variables was analyzed with Pearson’s chi-square test. Path analyses were conducted in Mplus [50], with maximum likelihood, ML estimation. 

**Figure 2:**
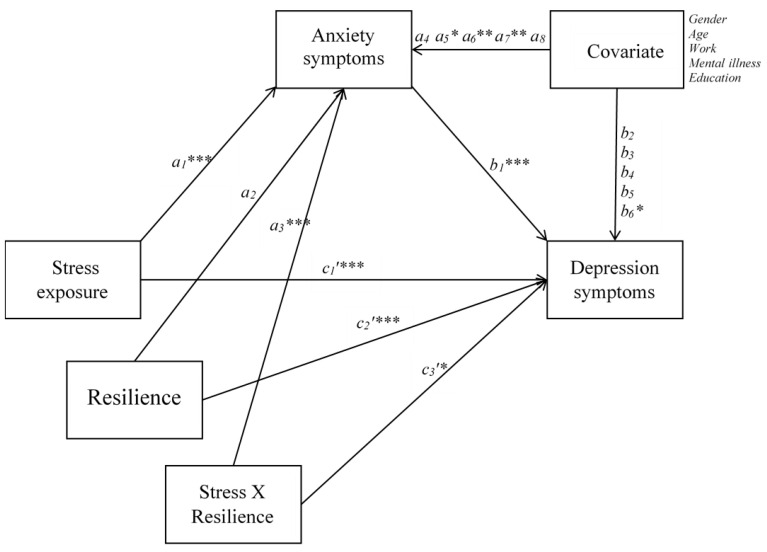
Path analysis of the moderated mediation model. * *p* < 0.05; ** *p* < 0.01; *** *p* < 0.001.

Hypothesis 1 was tested by estimating a mediation effect of anxiety symptoms in the relations between stress and depressive symptoms. When the mediation effect was significant, it was decided to proceed to test the second hypothesis. Resilience was introduced into the model as a moderator of both the direct effect, and the indirect effect of stress on depressive symptoms in a moderated mediation model (Figure 2). A significant mediating effect was established when the 95% bias-corrected bootstrap confidence interval based on 10,000 bootstrap draws did not contain zero. Similarly, evidence of moderated mediation was confirmed when the 95% bias-corrected bootstrap confidence interval for the index of moderated mediation [36] did not contain zero. We used a standard deviation above the mean, the mean, and a standard deviation below the mean to represent high, mean, and low subgroups of resilience. There was a total of 1.6% missing values on self-report measures, which were substituted with subscale averages. Relevant control variables included were age, gender (male, female, other), level of education (scale from 1 to 6, where higher score indicates higher level of education), working status (not working = 0, working or studying = 1), and history of mental disorder (no = 0; yes = 1).

## 3. Results

### 3.1. Preliminary Results

A total of 617 persons completed the survey. Of the total sample, 458 were female and 159 were male. Mean age was 38 (SD = 12.02). Please refer to Table 1 for sample characteristics. Table 2 shows means and standard deviations and for the included measures. For PHQ-9, 20.6% (*n* = 127) of the sample were above the 10-point cut-off of major depressive disorder (MDD) [49]. For GAD-7, 12% (*n* = 74) of the sample were above the cut-off of 10 which indicates an anxiety disorder [46]. Sixty-six (10.7%) scored above cut-off only on PHQ-9, *n* = 13 (2.1 %) scored above cut-off only on GAD-7, whereas *n* = 61 (9.9%) scored above cut-off on both PHQ-9 and GAD-7. Of the 210 respondents with a history of mental disorder, the results showed that 17% (*n* = 36) had a PHQ-9 score indicative of MDD, 3% (*n* = 7) had a GAD-7 score indicating an anxiety disorder, and 21% (*n* = 44) scored above cut-off on both PHQ-9 and GAD-7. The respondents (*n* = 210, 34%) who reported a history of mental disorder scored significantly lower on RSA t(615) = 8.226, *p* < 0.001) and significantly higher on PHQ-9 t(615) = −9.036, *p* < 0.001. In addition, persons with a history of mental illness had higher scores on PSS-14, t(615) = −8.035, *p* < 0.001 and GAD-7 t(615) = 8.985, *p* < 0.001. With respect to sex differences, men scored significantly higher than women on the RSA subscale Perception of self t(614) = 3.49, *p* = 0.001, whereas women scored significantly higher than men on Social resources, t(615) = −3.45, *p* = 0.001. Scores on PSS-14 were significantly higher for women than for men, t(615) = 3.642, *p* < 0.001. Symptoms of anxiety were significantly higher for women than for men, t(615) = 2.657, *p* < 0.01.

For those with a history of mental illness, *n* = 39 were out of work. Of these, *n* = 15 (2.4%) were on sick leave and *n* = 16 (2.6%) were on disability pension or out of work/at home. For those without a history of mental illness, *n* = 17 were out of work, and of these *n* = 2 (0.3%) were on sick leave, *n* = 8 (1.3%) received disability benefits and 1.1 % were out of work/at home. A chi-square test showed that significantly more respondents with a history of mental illness were out of work χ(1) = 34.80, *p* < 0.001.

### 3.2. Main Results

#### 3.2.1. Hypothesis 1

For the first hypothesis, we expected symptoms of anxiety to mediate the relations between exposure to stress and depressive symptoms. The results showed that the total effect of stress on depressive symptoms was significant and positive (standardized: β = 0.650, *p* < 0.001). Direct effect of stress was significantly positively associated with anxiety (β = 0.644, *p* < 0.001) and depressive symptoms (β = 0.357, *p* < 0.001). Direct effect of anxiety symptoms was significantly positively associated with depressive symptoms (β = 0.456, *p* < 0.001). Indirect effect of stress through anxiety symptoms on depressive symptom was significant and positive (β = 0.294, *p* < 0.001; [95% BCa: 0.234, 0.358]), supporting the first hypothesis. 

#### 3.2.2. Hypothesis 2

According to hypothesis 2 we expected that resilience would moderate both the direct and indirect effect of exposure to stress on depressive symptoms. In the moderated mediation model (Table 3), the interaction between stress and resilience was significant and negative on anxiety symptoms (β = −0.131, *p* < 0.001), and on depressive symptoms (β = −0.068, *p* < 0.05), indicating that resilience moderated the relations between stress and anxiety symptoms as well as between stress and depressive symptoms. However, evidence that the indirect effect of stress on depressive symptom was moderated by resilience (i.e., significant moderated mediation) was confirmed by the index of moderated mediation (IMM = −0.036, *p* < 0.001; [95% BCa: −0.055, −0.020]). Subgroup analyses conducted for the moderation of the direct relations between stress and depression indicated that high resilience (High resilience: HR = 0.120, *p* < 0.01), mean levels (mean resilience: MR = 0.166, *p* < 0.001), and low level of resilience (Low resilience: LR = 0.212, *p* < 0.01) were differentiated by their effects. Similarly, moderation of the indirect effect of stress on depressive symptoms indicated that high levels of resilience (HR = 0.114, *p* < 0.001), mean levels (MR = 0.151, *p* < 0.001), and low level of resilience (LR = 0.187, *p* < 0.001) were differentiated, supporting the second hypothesis. Thus, the high resilience subgroup was less affected than the low resilience subgroup by both the direct and indirect effect of stress associated with depressive symptoms.

## 4. Discussion

The present study investigated if resilience moderates negative outcome from stress during the COVID-19 pandemic. With respect to the primary hypothesis, the results showed that the relationship between stressful life events and symptoms of depression was mediated by anxiety symptoms. These findings are in line with previous research on the effects of stress on depression [38]. The results also supported the second hypothesis, with results suggesting the role of resilience as a moderator of stress-related depressive symptoms. Persons high on resilience reported lower levels of depression, compared to persons with mean or low degree of resilient factors. These results corroborate previous empirical data on the role of resilience as a moderator of negative outcome after exposure to stressful events [51].

The results demonstrate the importance of resilience resources with respect to how well an individual copes with stressful life circumstances, such as the COVID-19 pandemic. Importantly, this study shows that persons high on resilience were less affected by negative emotional reactions due to the experience of stress during the pandemic. These findings are similar to those reported during previous global virus outbreaks, like the SARS virus [31], which showed that resilient persons were less affected by negative reactions such as increased worry and anxiety.

To avoid response bias, COVID-19 was not explicitly mentioned in the survey, but the time frame for the different questionnaires covered the period after the COVID-19 outbreak. Thus, the questionnaires were completed during the initial outbreak of COVID-19 in Norway when the issue caused a great deal of worry and uncertainty. The present study may therefore be of relevance when studying the importance of resilience during national and global crisis and developing preventive actions. For a clinical interpretation, the results suggest that level of resilience is important for how well an individual copes with stress exposure. In terms of COVID-19 as a stressor, persons high on resilience are less affected with respect to negative outcomes like depression as well as the relationship between stress, anxiety and depression compared to those with low on resilience factors. However, the results did not indicate an increased level of emotional distress during the COVID-19 pandemic in Norway. To the contrary, the mean levels of depression and anxiety were equal to or somewhat lower than comparable studies with similar samples recruitment by convenience (e.g., [52,53]). With respect to level of stress, the mean PSS-14 score of the current sample was comparable to the scores originally reported in the original study [42]. Overall these results suggest that the reported levels of stress and emotional distress did not differ markedly compared to other Norwegian samples with a similar procedure for recruitment. 

The results of the moderated mediation analyses showed a significant direct effect of stress on depression and a significant indirect effect of stress on anxiety. This means that the effect of stress on depression is partly mediated by anxiety, meaning that persons prone to anxiety and worry are vulnerable for experiencing higher severity of symptoms of depression when exposed to stressors. The results show that the effect of resilience is differentiated for subgroups, implying that the degree of protection is dependent upon the level of resilience. The results show that when subgroups of resilience of high, medium and low resilience are taken into account, the relationship between stress and depression and the indirect relationship between stress, anxiety and depression is weaker for persons high on resilience compared to those with medium and low levels of resilience. From a societal perspective this may indicate that potential interventions of protection may be differentiated dependent on the characteristics of groups. The high, medium and low resilience groups may require different interventions or maybe interventions with different levels of intensity or magnitude. Such interventions may, as recommended previously, at the individual level include helping individuals to set measurable and realistic goals [33]. The community may thus tailor the promotion of resilience based on which subgroup the individual appears to belong.

The moderated-mediation analysis also revealed that age and employment status contributed significantly to the equation when included as control variables. However, this effect was only significant for anxiety symptoms. These findings suggest that higher age and being in work contribute to lower levels of anxiety, whereas being diagnosed with a mental illness was positively associated with level of anxiety symptoms. The results are in line with previous research which show the importance of work as a protective factor for developing depressive symptoms. Employment is related to better level of health and well-being [54]. Persons who are employed are also found to demonstrate less use of unhelpful coping strategies like avoidant behaviour and use of alcohol [55]. Our study adds to the literature that shows the beneficial effect of employment in terms of improved mental health and increased resilient behaviour.

The results also revealed gender differences in line with previous research [20,21]. For the RSA total score there were no gender difference, but for the RSA factors mean scores, men scored higher on Perception of self and women had higher score on Social resources. The former finding has been reported in previous research on resilience among adults [56]. The latter finding corroborates previous research on adults [20,26] and adolescents [28,31]. There seems to be a tendency for men to report a higher feeling of competency compared to women, whereas women describe themselves as lower on introversion, having more social support and more extended social networks [24]. These results have clinical relevance as they indicate which resources typically are preferred by women and men, and clinicians may bear in mind such possible sex differences when working with clients. 

There were also some important subgroup differences between those with and without a history of common mental disorders. There was a tendency for persons who reported mental disorders to report lower levels of resilience, which is in line with studies that show resilience to predict hopelessness [29]. These results may be of relevance for clinicians who work with individuals suffering from mental disorders. During times of heightened stress exposure like the COVID-19 situation, clinical therapists may help individuals to improve resilience skills. At the personal level this may include interventions to increase self-efficacy, and in terms of social resources this could imply working to increase the social network [57]. 

The current study suffers from limitations which may influence the interpretation of the results. The study is based on a cross-sectional sample, which means that the study is unable to answer questions regarding protective processes. This would require a longitudinal research design. Likewise, we cannot know if the self-report data truly reflects exposure to the COVID-19 situation, since this was not explicitly referred to in the survey. This may however also be a strength since any referral to the COVID-19 may have caused response bias regarding social desirability, as there may be different opinions about how to perceive and relate to the COVID-19 situation. The generalizability of the study is limited by convenience sampling, and future studies should apply random sampling of the general population. It should also be noted that the COVID-19 situation in Norway is not necessarily generalizable to other countries. However, the results were comparable to previous Norwegian studies of similar designs, which supports the generalizability of the results in general. The reported history of mental disorder was based on self-report and not a clinical evaluation, which limits the generalizability to groups of patients diagnosed with mental disorders.

## 5. Conclusions

The present study investigated a moderated mediation model of the association between exposure to stress and depressive symptoms. The results showed that resilience moderated the relations between stress and anxiety symptoms as well as between stress and depressive symptoms, and symptoms of anxiety mediated the association between stress and depressive symptoms. Individuals with high level of resilience were less affected than individuals with low level of resilience. 

## Figures and Tables

**Table 1:** Sample characteristics.

Demographics	*n*	%
**Sex**		
Male	159	25.8
Female	458	74.2
**Marital status**		
Married/partner	435	70.5
Single	176	28.5
Widowed	6	1
**Mental illness**	210	34
**Employment**		
Working	437	70.8
Student	113	18.3
Sick leave	17	2.8
<1 year	11	1.8
>1year	6	1.0
Disability pension	24	3.9
Out of work/at home	15	2.4
Retired	11	1.8
**Education**		
Primary/secondary school	10	1.6
High school (1–2 years)	17	2.8
High school (3 years)	59	9.6
Technical school	36	5.8
College/university (<4 years)	171	27.7
College/university (>4 years)	324	52.5

Mental illness = Participants confirming a lifetime history of mental disorder.

**Table 2:** Means, standard deviations and correlation coefficients for the study variables.

	Variable	Mean	SD	2	3	4	5	6	7	8	9
1	Stress	23.72	7.61	0.73 ^b^	0.72 ^b^	−0.67 ^b^	15 ^b^	−0.24 ^b^	−0.17 ^b^	0.30 ^b^	−0.25 ^b^
2	Anx	4.70	4.10		0.75 ^b^	−0.56 ^b^	0.12 ^b^	−0.23 ^b^	−0.19 ^b^	0.34 ^b^	−0.24 ^b^
3	Dep	5.99	4.93			−0.64 ^b^	0.07	−0.18 ^b^	−0.22 ^b^	0.32 ^b^	−0.32 ^b^
4	Resilience	5.16	0.89				0.01	0.13 ^b^	0.22 ^b^	−0.31 ^b^	0.28 ^b^
5	Gender							−0.08 ^a^	−0.03	0.07	0.06
6	Age	38.00	12.02						−0.21 ^a^	−0.08	0.15 ^b^
7	Work									−0.21 ^b^	0.19 ^b^
8	Mental illness										0.08 ^a^
9	Education	5.13	1.21								

Stress = PSS-14. Anx = GAD-7. Dep = PHQ-9. Resilience = RSA total score. Work = In or out of work or retired. Mental illness = History of mental illness (yes). Educatio*n* = Level of education. ^a^ *p* < 0.05. ^b^ *p* < 0.01. Spearmans rho utilized for correlations with gender, work, mental illness and education.

**Table 3:** Path coefficients from the moderated mediation model.

	Outcome Variables					
Path	Anxiety Symptoms			Depressive Symptoms		
		*β*	95%		*β*	95%
Stress→	*a_1_*	0.583 ***	[0.506, 0.657]	*c_1_’*	0.256 ***	[0.171, 0.344]
Anxiety symptoms→		-		*b_1_*	0.399 ***	[0.306, 0.492]
Resilience→	*a_2_*	−0.055	[−0133, 0.021]	*c_2_’*	−0.196 ***	[−0.282, −0.106]
Stress X Resilience→	*a_3_*	−0.131 ***	[−0.186, −0.073]	*c_3_’*	−0.068 *	[−0.129, −0.002]
Gender→	*a_4_*	0.002	[−0.058, 0.050]	*b_2_*	−0.037	[−0.087, 0.012]
Age→	*a_5_*	−0.086 *	[−0.138, −0.032]	*b_3_*	0.012	[−0.032, 0.060]
Work→	*a_6_*	−0.097 **	[−0.165, −0.032]	*b_4_*	−0.025	[−0.087, 0.039]
Mental→	*a_7_*	0.096 **	[0.033, 0.156]	*b_5_*	0.043	[−0.013, 0.100]
Education→	*a_8_*	−0.014	[−0.075, 0.047]	*b_6_*	−0.055	[−0.116, 0.001]
		*R*^2^ = 0.581			*R*^2^ = 0.668	

*β* = Standardized path coefficients; * *p* < 0.05; ** *p* < 0.01; *** *p* < 0.001.

## References

[1] Brooks S.K., Webster R.K., Smith L.E., Woodland L., Wessely S., Greenberg N., Rubin G.J. (2020). The Psychological Impact of Quarantine and How to Reduce It: Rapid Review of the Evidence. Lancet.

[2] Korstanje M.E. (2011). Swine flu in Buenos Aires: Beyond the principle of resilience. Int. J. Disaster Resil. Built Environ..

[3] Nebehay S., Farge E. (2020). Coronavirus Emergency Is ‘Public Enemy Number 1’. WHO via Reuters.

[4] Taylor S., Asmundson G.J.G. (2004). Treating Health Anxiety: A Cognitive Behavioral Approach.

[5] Wong T.W., Gao Y., Tam W.W.S. (2006). Anxiety among university students during the SARS epidemic in Hong Kong. Stress Health.

[6] Maunder R., Hunter J., Vincent L., Bennett J., Peladeau N., Leszcz M., Sadavoy J., Verhaeghe L.M., Steinberg R., Mazzulli T. (2003). The immediate psychological and occupational impact of the 2003 SARS outbreak in a teaching hospital. Can. Med. Assoc. J..

[7] Lau J.T., Kim J.H., Tsui H.Y., Griffiths S. (2008). Perceptions Related to Bird-to-Human Avian Influenza, Influenza Vaccination, and Use of Face Mask. Infection.

[8] Wheaton M.G., Abramowitz J.S., Berman N.C., Fabricant L.E., Olatunji B.O. (2011). Psychological Predictors of Anxiety in Response to the H_1_N_1_ (Swine Flu) Pandemic. Cogn. Ther. Res..

[9] Bish A., Michie S. (2010). Demographic and attitudinal determinants of protective behaviours during a pandemic: A review. Br. J. Health Psychol..

[10] Lazarus S.R., Folkman S. (1984). Stress, Appraisal, and Coping.

[11] Mohaupt S. (2009). Resilience and social exclusion. Soc. Policy Soc..

[12] Egeland B., Carlson E., Sroufe L.A. (1993). Resilience as process. Dev. Psychopathol..

[13] Rutter M. (1985). Resilience in the Face of Adversity: Protective factors and resistance to psychiatric disorder. Br. J. Psychiatry.

[14] Luthar S.S., Cicchetti D., Cohen D.J. (2006). Resilience in development: A synthesis of research across five decades. Developmental Psychopathology: Risk, Disorder, and Adaptation.

[15] Masten A.S., Obradović J. (2008). Disaster Preparation and Recovery: Lessons from Research on Resilience in Human Development. Ecol. Soc..

[16] Rutter M. (2012). Resilience as a dynamic concept. Dev. Psychopathol..

[17] Gallo C.L., Matthews K.A. (2003). Understanding the association between socioeconomic status and physical health: Do negative emotions play a role?. Psychol. Bull..

[18] Fletcher D., Sarkar M. (2013). Psychological Resilience: A review and critique of definitions, concepts, and theory. Eur. Psychol..

[19] Chmitorz A., Kunzler A., Helmreich I., Tüscher O., Kalisch R., Kubiak T., Wessa M., Lieb K. (2018). Intervention studies to foster resilience—A systematic review and proposal for a resilience framework in future intervention studies. Clin. Psychol. Rev..

[20] Friborg O., Hjemdal O., Rosenvinge J.H., Martinussen M. (2003). A new rating scale for adult resilience: What are the central protective resources behind healthy adjustment?. Int. J. Methods Psychiatry Res..

[21] Hjemdal O., Friborg O., Martinussen M., Rosenvinge J.H. (2001). Preliminary results from the development and validation of a Norwegian scale for measuring adult resilience. J. Nor. Psychol. Assoc..

[22] Friborg O., Barlaug D., Martinussen M., Rosenvinge J.H., Hjemdal O. (2005). Resilience in relation to personality and intelligence. Int. J. Methods Psychiatry Res..

[23] Hjemdal O. (2007). Measuring Protective Factors: The Development of Two Resilience Scales in Norway. Child Adolesc. Psychiatr. Clin. N. Am..

[24] Feingold A. (1994). Gender differences in personality: A meta-analysis. Psychol. Bull..

[25] Cross E.S., Markus H.R., Beall A.E., Sternberg R.J. (1993). Gender in thought, belief, and action: A cognitive approach. The Psychology of Gender.

[26] Werner E.E. (1989). High-risk children in young adulthood: A longitudinal study from birth to 32 years. Am. J. Orthopsychiatry.

[27] Hyde J.S. (2005). The gender similarities hypothesis. Am. Psychol..

[28] Hjemdal O., Friborg O., Stiles T.C., Rosenvinge J.H., Martinussen M. (2006). Resilience predicting psychiatric symptoms: A prospective study of protective factors and their role in adjustment to stressful life events. Clin. Psychol. Psychother..

[29] Hjemdal O., Friborg O., Stiles T.C. (2011). Resilience is a good predictor of hopelessness even after accounting for stressful life events, mood and personality (NEO-PI-R). Scand. J. Psychol..

[30] Pietrzak R.H., Johnson D.C., Goldstein M.B., Malley J.C., Southwick S.M. (2009). Psychological resilience and postdeployment social support protect against traumatic stress and depressive symptoms in soldiers returning from Operations Enduring Freedom and Iraqi Freedom. Depress. Anxiety.

[31] Hjemdal O., Vogel P.A., Solem S., Hagen K., Stiles T.C. (2010). The relationship between resilience and levels of anxiety, depression, and obsessive-compulsive symptoms in adolescents. Clin. Psychol. Psychother..

[32] Bonanno G.A., Ho S.M., Chan J.C.K., Kwong R.S.Y., Cheung C.K.Y., Wong C.P.Y., Wong V.C.W. (2008). Psychological resilience and dysfunction among hospitalized survivors of the SARS epidemic in Hong Kong: A latent class approach. Health Psychol..

[33] Rosenberg A.R. (2020). Cultivating Deliberate Resilience During the Coronavirus Disease 2019 Pandemic. JAMA Pediatrics.

[34] Anyan F., Bizumic B., Hjemdal O. (2017). Specificity in mediated pathways by anxiety symptoms linking adolescent stress profiles to depressive symptoms: Results of a moderated mediation approach. J. Affect. Disord..

[35] Hayes A.F. (2013). Mediation, Moderation, and Conditional Process Analysis.

[36] Hayes A.F. (2015). An Index and Test of Linear Moderated Mediation. Multivar. Behav. Res..

[37] Preacher K.J., Rucker D.D., Hayes A.F. (2007). Addressing Moderated Mediation Hypotheses: Theory, Methods, and Prescriptions. Multivar. Behav. Res..

[38] Anyan F., Worsley L., Hjemdal O. (2017). Anxiety symptoms mediate the relationship between exposure to stressful negative life events and depressive symptoms: A conditional process modelling of the protective effects of resilience. Asian J. Psychiatry.

[39] Wang C., Pan R., Wan X., Tan Y., Xu L., Ho C.S.H., Ho R. (2020). Immediate Psychological Responses and Associated Factors during the Initial Stage of the 2019 Coronavirus Disease (COVID-19) Epidemic among the General Population in China. Int. J. Environ. Res. Public Health.

[40] Holmes E.A., O’Connor R.C., Perry V.H., Tracey I., Wessely S., Arseneault L., Ballard C., Christensen H., Silver R.C., Everall I. (2020). Multidisciplinary research priorities for the COVID-19 pandemic: A call for action for mental health science. Lancet Psychiatry.

[41] Brand J.E. (2015). The Far-Reaching Impact of Job Loss and Unemployment. Annu. Rev. Sociol..

[42] Cohen S., Kamarck T., Mermelstein R., Mermelstein T.K. (1983). A Global Measure of Perceived Stress. J. Health Soc. Behav..

[43] Lee E.-H. (2012). Review of the Psychometric Evidence of the Perceived Stress Scale. Asian Nurs. Res..

[44] Walvekar S.S., Ambekar J.G., Devaranavadagi B.B. (2015). Study on Serum Cortisol and Perceived Stress Scale in the Police Constables. J. Clin. Diagn. Res..

[45] Friborg O., Hjemdal O., Rosenvinge J.H., Martinussen M., Aslaksen P.M., Flaten M.A. (2006). Resilience as a moderator of pain and stress. J. Psychosom. Res..

[46] Spitzer R.L., Kroenke K., Williams J.B.W., Löwe B. (2006). A Brief Measure for Assessing Generalized Anxiety Disorder. Arch. Intern. Med..

[47] Beard C., Björgvinsson T. (2014). Beyond generalized anxiety disorder: Psychometric properties of the GAD-7 in a heterogeneous psychiatric sample. J. Anxiety Disord..

[48] Johnson S.U., Ulvenes P.G., Oktedalen T., Hoffart A. (2019). Psychometric properties of the GAD-7 in a heterogeneous psychiatric sample. Front. Psychol..

[49] Kroenke K., Spitzer R.L., Williams J.B.W. (2001). The PHQ-9—Validity of a brief depression severity measure. J. Gen. Intern. Med..

[50] Muthén K.L., Muthén B.O. (2017). Mplus: Statistical Analysis with Latent Variables (Version 7.4).

[51] Anyan F., Hjemdal O. (2016). Adolescent stress and symptoms of anxiety and depression: Resilience explains and differentiates the relationships. J. Affect. Disord..

[52] Hagen K., Solem S., Opstad H.B., Hansen B., Hagen R. (2017). The role of metacognition and obsessive-compulsive symptoms in psychosis: An analogue study. BMC Psychiatry.

[53] Solem S., Thunes S.S., Hjemdal O., Hagen R., Wells A. (2015). A Metacognitive Perspective on Mindfulness: An Empirical Investigation. BMC Psychol..

[54] Friedland D.S., Price R.H. (2003). Underemployment: Consequences for the health and well-being of workers. Am. J. Community Psychol..

[55] Perreault M., Touré E.H., Perreault N., Caron J. (2016). Employment Status and Mental Health: Mediating Roles of Social Support and Coping Strategies. Psychiatr. Q..

[56] Hjemdal O., Friborg O., Braun S., Kempenaers C., Linkowski P., Fossion P. (2011). The Resilience Scale for Adults: Construct Validity and Measurement in a Belgian Sample. Int. J. Test..

[57] Iacoviello B.M., Charney D.S. (2014). Psychosocial facets of resilience: Implications for preventing posttrauma psychopathology, treating trauma survivors, and enhancing community resilience. Eur. J. Psychotraumatol..

